# “Non-dipping” is equally frequent in narcoleptic patients and in patients with insomnia

**DOI:** 10.1007/s41105-015-0004-z

**Published:** 2015-11-26

**Authors:** Mariusz Sieminski, Markku Partinen

**Affiliations:** Department of Adults’ Neurology, Medical University of Gdansk, Debinki 7, 80-210 Gdansk, Poland; Vitalmed Helsinki Sleep Clinic, Sitratori 3, 00420 Helsinki, Finland

**Keywords:** Narcolepsy, Cataplexy, Blood pressure, “Non-dipping”

## Abstract

Narcolepsy with cataplexy (NC) is a neurological sleep disorder characterized by very low or undetectable concentration of hypocretin-1 in the cerebrospinal fluid. It has been recently found that patients with NC have disturbed circadian pattern of blood pressure, with more frequent non-dipping, compared to healthy controls. It has been hypothesized that lack of hypocretin may lead to increase in nocturnal blood pressure. This increase may result also from disturbed sleep architecture regardless of the deficiency of hypocretin. The aim of this study was to compare changes in values of daytime and nighttime blood pressure in NC patients and in patients with disturbed nocturnal sleep due to other sleep disorders. We have retrospectively compared polysomnographic and clinical data of 8 NC patients and 7 age- and sex controls suffering from insomnia. We have compared sleep architecture, mean blood pressure values and dipping pattern in both groups. The groups did not differ in terms of disturbances of sleep architecture. We have not found any statistical differences in values of daytime and nocturnal blood pressure. Non-dipping was equally frequent in both groups (87.5 and 85.7 %). Our results suggest that observed abnormalities in circadian changes of blood pressure values result from disturbed sleep architecture than from deficiency of hypocretin. Patients with sleep disorders should be carefully observed for the presence of increased blood pressure and other vascular risk factors.

## Introduction

Narcolepsy with cataplexy (NC) is a neurological sleep disorder characterized by the presence of excessive daytime sleepiness, cataplexy, hypnagogic hallucinations and sleep paralysis. Nocturnal sleep disturbances and automatic behaviors during the day are also present in the disease [1]. It was found that narcoleptic patients are characterized by very low or undetectable concentration of hypocretin-1 in the cerebrospinal fluid. Hypocretin-1 (Hcrt-1) is a neurotransmitter produced by neurons located in the lateral part of hypothalamus, projecting to multiple areas of the central nervous system and influencing the sleep–wake rhythm and many other metabolic processes [2]. It has been also hypothesized that hypocretin is a relevant factor in central control of the circadian rhythm of fluctuations of the cardiovascular system [3, 4].

One of the most prominent traits of the circadian rhythm of the cardiovascular system is the nocturnal decrease of the blood pressure (BP)—so-called dipping. This phenomenon has a crucial clinical significance—decrease in nocturnal values of blood pressure that below 10 % of the daytime values (so-called non-dipping) or increase of the BP during the night (“inverse dipping”) are major cardiovascular risk factors [5].

Non-dipping pattern of nocturnal blood pressure was recently found in NC patients in two studies [6, 7]. The authors comparing NC patients with healthy controls found that the nocturnal decrease of blood pressure is reduced in NC patients [6] or that non-dipping is more prevalent in narcoleptics [7]. The authors of both the studies hypothesized that it is the deficit of hypocretin, which causes this abnormalities in narcoleptic patients.

Increased cardiovascular risk or non-dipping has been found also in other sleep disorders, like insomnia [8]. It might be hypothesized that lack of nocturnal dipping of blood pressure in narcoleptic patients is not resulting from deficit of hypocretin but that it is secondary to disturbed sleep architecture.

The aim of this study was to compare changes in values of daytime and nighttime blood pressure in NC patients and in patients with disturbed nocturnal sleep due to insomnia.

## Materials and methods

We have performed a retrospective analysis of polysomnographic, laboratory and clinical data of 8 patients diagnosed in VitalMed Helsinki Sleep Clinic with NC. All the patients were diagnosed according to the International Classification of Sleep Disorders (ICSD-2) [9]. We have analyzed the first polysomnographic recording of drug-naïve patients, performed during the diagnostic process, without intake of any drugs (including anti-narcoleptic drugs) that might have interfered with the sleep architecture. Data on hypocretin-1 (hcrt-1) level in the cerebrospinal fluid and the on the haplotype HLA DQB1*0602 were collected. Data on the comorbid disorders and drug intake were taken from the patients files.

We have randomly selected 7 sex- and age-matched controls from the patients that underwent a diagnostic polysomnography (PSG) in VitalMed Helsinki Sleep Clinic and were diagnosed with insomnia. We have excluded patients diagnosed with sleep-related breathing disorders (SRBD) as the influence of SRBD on the function of cardiovascular system is very significant and well described. As the NC patients, controls were free of taking any drugs that might have altered their sleep architecture during the PSG. Their clinical and laboratory data (including data on hypocretin-1 level and haplotype HLA DQB1*0602, where available) were taken from their medical files.

Data of all the patients were anonymized prior to selection of the patients. Only archival data of the patients were analyzed. The protocol of the study was approved by the Independent Bioethical Committee for Scientific Research at the Medical University of Gdansk.

Patients underwent a single polysomnographic study. All the recordings were performed with SOMNOscreen plus PSG system (Somnomedics, Randersacker, Germany). Sleep recording included four EEG leads, two bilateral electro-oculogram leads (EOG), bilateral chin electromyographic leads (EMG), two surface EMG leads placed on the left and right anterior tibialis muscles (recording periodic limb movements (PLMs) in sleep and wake). Respiration was recorded with nasal cannula, thoracic and abdominal strains and finger oxymetry. ECG was recorded with single precardial lead. The PSG included beat-to-beat blood pressure measurement that was performed with measurement of pulse transit time (PTT) [10]. The measurement of blood pressure was continuous, non-invasive and not disturbing the sleep of the patients.

Recordings were scheduled to start at 09.00 PM and to end at 06.30 AM, with 9 h and 30 min of recording. The patients were allowed to sustain their normal activity and were not forced to stay in bed for at least 1 h after beginning of the recording.

The PSG recordings were scored according to American Academy of Sleep Medicine guidelines [11]. The following sleep parameters were calculated: total sleep time (TST); sleep efficiency (SE); latency of stages 1, 2, slow wave sleep (SWS) and REM sleep; duration of stages 1, 2, SWS and REM; Sleep Stage Change Index (number of transitions between the sleep stages per hour of sleep); the Wake Index (WI) (number of awakenings per hour of sleep), duration of Wake after Sleep Onset (WASO), PLMS index (PLMSI).

For the assessment of the cardiovascular system the following values were calculated: mean systolic and diastolic blood pressure (SBP and DBP, respectively) during the “day” (“day” was defined as the time from the start of recording till the “lights-off” moment, and from the “lights-on” moment till the end of recording) and during the night (defined as the time between “lights-off” and “lights-on” moments), the difference between “day” and night mean values of the SBP and DBP (delta_SBP and delta_DBP) and the percentage of the BP values reduction during the night (delta_SBP/SBP_day × 100 %; delta_DBP/DBP_day × 100 %). Patients with decrease of mean blood pressure (systolic or diastolic) during the night smaller than 10 % of mean blood pressure during the “day” were considered as “non-dippers”. Mean heart rate (HR) during the “day” and the night and the “day”-night HR difference (delta_HR) were also calculated. Sympatho-vagal balance (SVB) was assessed by calculating an average ratio between low (LF) and high frequency (HF) compononents of heart rate variability (HRV) spectra.

## Results

Recordings of 8 NC patients and 7 insomnia patients were analyzed. The demographic and clinical data of the patients are presented in Table 1. There was 1 hypertensive patient in the narcolepsy and in the insomnia group. None of them was medicated when the PSG was performed.

**Table 1:** Demographic and clinical data of the studied population

	NC patients (*n* = 8)	Controls (*n* = 7)	*P*
Gender (M/F)	3/5	4/3	NS
Age (years, mean ± SD)	27.88 ± 15.39	24.29 ± 6.80	NS
BMI (mean ± SD)	25.44 ± 5.75	26.6 ± 5.36	NS
ESS (mean ± SD)	15.88 ± 4.55	9.14 ± 4.67	<0.005
Comorbidities			
Hypertension [*n* (%)]	1 (12.5)	1 (14.3)	NS
Hypercholesterolaemia [*n* (%)]	1 (12.5)	1 (14.3)	NS
Cluster headache [*n* (%)]	1 (12.5)	0 (0)	NS
Current smoking [*n* (%)]	4 (50)	4 (57.1)	NS

*BMI* body mass index, *ESS* Epworth sleepiness scale

Data on hypocretin-1 level in cerebrospinal fluid were available for all NC patients. Hcrt-1 was undetectable in 5 cases, and its concentration ranged from 27.00 to 69.00 in 3 cases. Data on haplotype HLA DQB1*0602 were collected in 8 patients—all of them were positive. Data on haplotype HLA DQB1*0602 and Hcrt-1 level in cerebrospinal fluid were available for 4 control patients—all of them were negative for HLA DQB1*0602 and had Hcrt-1 level above 110 pg/ml.

There were no statistically significant differences in the sleep parameters between the groups with an exception for longer REM latency in the NC group. The sleep parameters of the NC patients and the control patients are presented in Table 2.

**Table 2:** Sleep parameters of NC patients and controls

	NC patients	Insomnia patients	*P*
Total sleep time (min; mean ± SD; median)	442.8 ± 70.2; 462.6	427.2 ± 67.8; 433.8	0.34
Sleep efficiency (%)	84.41 ± 11.86	85.94 ± 11.13	0.40
S1 latency (min; mean ± SD; median)	15.6 ± 26.4; 6.0	33.0 ± 47.4; 21.0	0.19
S2 latency (min; mean ± SD; median)	32.4 ± 24.6; 22.8	42.6 ± 48.6; 42.6	0.81
SWS latency (min; mean ± SD; median)	40.2 ± 22.8; 30.6	63.0 ± 67.8; 37.8	0.19
REM latency (min; mean ± SD; median)	15.6 ± 31.2; 4.2	106.8 ± 54.0; 101.4	0.0006
S1 time (min;; mean ± SD; median)	99.0 ± 35.4; 106.2	77.4 ± 97.2; 52.2	0.28
S2 time (min; mean ± SD; median)	186.0 ± 42.6; 187.8	210.0 ± 90.0; 241.2	0.25
SWS time (min; mean ± SD; median)	73.8.0 ± 37.8; 55.2	61.2 ± 40.2; 58.8	0.27
REM time (min; mean ± SD; median)	84.6 ± 18.6; 79.8	78.6 ± 25.8; 73.2.	0.32
Sleep stage change index (mean ± SD)	18.93 ± 6.81	15.43 ± 4.45	0.13
Wake index (mean ± SD)	3.58 ± 2.64	3.01 ± 2.01	0.33
Wake after sleep onset (min; mean ± SD; median)	65.4 ± 58.2; 45.0	32.4 ± 19.2; 35.4	0.09
PLMS index (mean ± SD)	7.44 ± 6.01	2.79 ± 4.69	0.06

Sleep stage change index: number of transitions between the sleep stages per hour of sleep; wake index: number of awakenings per hour of sleep, “Wake” defined as appearance of at least one epoch of sleep stage “Wake” from any sleep stage

There were no statistically significant differences between the groups regarding mean values of blood pressure and heart rate during “day” and night. The average sympatho-vagal balance did not differ significantly between the groups (Table 3).

**Table 3:** Mean values of blood pressure, heart rate and sympatho-vagal balance compared between narcolepsy and insomnia groups

	NC patients	Insomnia patients	*P*
Mean “day” SBP ± SD	115.7 ± 12.1	116.14 ± 13.2	0.48
Mean night SBP ± SD	110.2 ± 9.5	107.4 ± 9.9	0.29
Mean “day” DBP ± SD	71.7 ± 7.9	70.6 ± 6.1	0.37
Mean night DBP ± SD	69.4 ± 7.2	65.7 ± 5.2	0.14
Mean “day” HR ± SD	75.9 ± 11.9	69.0 ± 14.5	0.16
Mean night HR ± SD	60.0 ± 3.8	60.7 ± 8.5	0.42
Mean SVB ± SD	14.8 ± 5.8	12.4 ± 2.4	0.16

*SBP* systolic blood pressure, *DBP* diastolic blood pressure, *HR* heart rate, *SVB* sympatho-vagal balance

There was a trend for less-pronounced nocturnal dipping in the narcoleptic group although the difference did not reach the statistical significance. The numbers of dippers were equal in both groups (Table 4).

**Table 4:** Mean values of nocturnal dipping of blood pressure and prevalence of non-dipping in narcoleptics (NC) and insomnia patients

	NC patients (*n* = 8)	Insomnia patients (*n* = 7)	*P*
Delta_SBP (mmHg)	5.5	8.7	0.23
Dipping of SBP (%)	4.4	7.0	0.23
Delta_DBP (mmHg)	2.4	4.9	0.09
Dipping of DBP (%)	3.1	6.7	0.07
Number of non-dippers	7	6	1.00

*Delta_SBP* decrease of systolic blood pressure during the night, *Delta_DBP* decrease of diastolic blood pressure during the night

## Discussion

Nocturnal dipping of blood pressure is an important physiologic phenomenon and non-dipping is a relevant risk factor for cardiovascular diseases. That is why clarifying the mechanisms of dipping has a crucial meaning. We have found that narcoleptic patients do not differ statistically from patients with insomnia in terms of values of blood pressure and presence of non-dipping. The prevalence of non-dipping was very high in both groups but did not differ between them. The control group (patients with insomnia) did not differ from the narcoleptic group in terms of demographic traits. Both groups had similarly disturbed nocturnal sleep. The only difference between the groups was the etiology of sleep disorders: presence or absence of hypocretin deficiency. The fact that we did not find any differences in values of blood pressure and in the values of nocturnal dipping of blood pressure between the groups suggests that the hypocretin status does not influence the cardiovascular parameters. High prevalence of “non-dippers” in both groups suggest that the disorders of sleep architecture, regardless of their etiology, may play a significant role in increasing the cardiovascular risk.

The relation between narcolepsy and blood pressure has been studied in animal models and the results are ambiguous. Schwimmer et al. found that blood pressure in narcoleptic rats is lower than in wild-type animals, which present decrease of the values of blood pressure during transition from wake to NREM sleep [4]. Shirasaka et al. found that hypocretin given intracerebroventricularly to conscious rats led to increase in the values of mean arterial pressure in the animals [12]. A similar conclusion was drawn after experimental injections of hypocretin to rostral ventrolateral medulla of anesthetized rats [13] or to nucleus tractus solitarius of anesthetized rats [14]. Matsumura et al. found that intracerebroventricular injection of hypocretin leads to increase of blood pressure in conscious rabbits [15]. Microinjections of hypocretin to medullary raphe increased heart rate and blood pressure in awake rats [16]. Recently it has been shown that blockade of central hypocretin receptors decreases blood pressure in hypertensive rats [17]. Those results suggest that in case of deficiency of hypocretin within the central nervous system an increase of blood pressure should not be expected. Our finding that blood pressure in narcoleptic patients is not higher than in patients without narcolepsy remains in line with the above conclusions. De Oliveira et al. found that the cardiovascular reaction (increase or decrease of the blood pressure) to microinjections of hypocretin depends on the site of the injection within the nucleus of solitary tract in rats [18]. There is one study so far showing that sleep-related dipping of blood pressure is blunted and that blood pressure during sleep is higher in hypocretin-deficient mice compared with wild-type animals [19].

Data on circadian shifts of blood pressure in patients with narcolepsy-cataplexy are scarce and unambiguous. Guilleimnault et al. compared nocturnal blood pressure in narcoleptic patients and healthy controls [20]. No significant differences were found in the values of blood pressure and the rhythm of their changes during the night between the groups, which is in concordance with our study. Baker et al. have published a study with examined populations similar to ours: narcoleptic patients were compared with patients suffering from another sleep–wake disorder—idiopathic hypersomnia [21]. The authors found that NC patients had higher values of nocturnal blood pressure than patients with hypersomnia. This result is opposite to ours. It must be noted that populations compared by Baker et al. differed significantly in terms of sleep parameters. Narcoleptic patients had significantly more apneic/hypopneic episodes and periodic limb movements during the night than patients with hypersomnia, they also had shorter total sleep time, less slow-wave sleep, more frequent awakenings and lower sleep efficiency. Any of those differences might have influenced the values of blood pressure. The populations compared in our paper, despite the diversity of diagnoses, had similar sleep parameters that may explain the discrepancy in the results between studies of Baker et al. and ours.

Narcoleptic patients in our study had higher values in Epworth Sleepiness Scale implicating higher level of daytime sleepiness. That could result in lower intensity of physical activity, which in turn could lead to lower values of blood pressure. That may partly explain the lack of significant difference between the daytime and nighttime values of blood pressure in the narcoleptic patients.

Recently two studies on blood pressure and nocturnal dipping in narcolepsy were published. Grimaldi et al. in a study comparing 10 narcoleptic patients and 12 healthy controls found significantly blunted decrease in blood pressure in NC patients compared with the controls. Narcoleptic patients in our study had lower values of decrease in blood pressure (Delta_SBP and Delta_DBP) but this difference did not reach statistical significance. Grimaldi et al. were comparing narcoleptics and healthy subjects [6]. There were some sleep parameters that significantly differed between the groups: arousal index, periodic limbs movements in sleep index and periodic limb movements in sleep with arousal index were significantly higher in the narcoleptic patients and their sleep was more fragmented. The authors stated that the blunted blood pressure dipping may result from disrupted sleep architecture as well as from deficiency of hypocretin. Groups compared in our study did not differ statistically in terms of sleep parameters (as two groups with sleep/wake disorders were compared) and this may explain the lack of difference in nocturnal dipping. Dauvilliers et al. performed a study comparing 50 NC patients with 42 healthy controls [7]. The authors found that dipping of diastolic and mean blood pressure was significantly lower in the NC group, with significantly higher percentage of “non-dippers” in the NC group. The groups differed significantly as sleep parameters were considered: percentage of REM sleep was higher in the NC group, latency of REM sleep was shorter in NC sleep, wake time after sleep onset, apnea hypopnea index, periodic limb movements index were greater in the NC group. The authors found associations between disturbed sleep architecture in narcoleptic patients and the dipping of the blood pressure and concluded that observed abnormalities in blood pressure dipping may result from sleep disturbances typical for narcolepsy. This suggestion was confirmed in our study. Our groups did not differ in terms of sleep parameters and no difference in blood pressure dipping was found.

Our study has some limitations. It was a study with a small number of patients and the control group was not homogenous, which may weaken the conclusions. We were focused on the phenotype of the disordered sleep and not on the aetiology of the sleep disorder. That enabled us to notice that non-dipping may be present in sleep-disordered subjects, regardless of the pathomechanism of the sleep disorder. The retrospective character of the study allowed for precise inclusion of the patients and controls that makes conclusions more reliable. Data on daytime blood pressure do not come from 24-h monitoring of blood pressure. Values of daytime blood pressure were acquired from early evening and early morning hours—period of less intense activity. Due to that BP values could have been reduced and thus influencing the values of dipping. Nevertheless, the patients were sustaining their normal activity after beginning of the recording. It must be also remembered that stay at the sleep laboratory and a PSG study was a completely new experience for the patients leading to some form of arousal. Thus the recorded BP values contain information about daytime BP values of the patients. As this limitation applies for both compared groups, the conclusions still may be drawn. Another limitation is lack of healthy control group—absence of non-dipping in patients with normal sleep would be another evidence for hypothesis that non-dipping may result from sleep disturbances.

The strength of our study is the selection of patients. We were analyzing PSG recordings of drug-naïve and drug-free NC patients that allows to exclude any influence of stimulating drugs on the blood pressure values. Both groups did not differ in terms of sleep parameters. The clinical phenotype of disturbances in sleep architecture was similar in both groups but the etiology of those disturbances was different. This allows us to conclude that lack of nocturnal dipping of blood pressure is frequent in narcoleptic patients but is not more frequent than in patients with other sleep disorder (insomnia). That suggests that it is not the deficiency of hypocretin that leads to abnormalities in nocturnal blood pressure but the disruption of sleep architecture, regardless of its etiology although due to small number of study participants it is a very preliminary conclusion. An interesting way of verifying this hypothesis in the future would be comparing values of nocturnal dipping of BP in narcoleptic patients with and without hypocretin deficiency. That requires a more strict assessment of cardiovascular risk in the whole clinical population of sleep-disordered patients.
